# Effects of cow reproductive status, parity and lactation stage on behaviour and heavy breathing indications of a commercial accelerometer during hot weather conditions

**DOI:** 10.1007/s00484-023-02496-2

**Published:** 2023-05-29

**Authors:** Lisette M.C. Leliveld, Daniela Lovarelli, Alberto Finzi, Elisabetta Riva, Giorgio Provolo

**Affiliations:** 1 Department of Agricultural and Environmental Sciences, University of Milan, via G. Celoria 2, 20133 Milan, Italy; 2 Department of Environmental Science and Policy, University of Milan, via G. Celoria 2, 20133 Milan, Italy

**Keywords:** Dairy cattle, Parity, Precision livestock farming, Panting, Cow welfare monitoring

## Abstract

Heat stress presents one of the most urgent challenges to modern dairy farming, having major detrimental impacts on cow welfare, health, and production. Understanding the effect of cow factors (reproductive status, parity, and lactation stage) on the physiological and behavioural response to hot weather conditions is essential for the accurate detection and practical application of heat mitigation strategies. To study this, collars with commercial accelerometer-based sensors were fitted on 48 lactation dairy cows to record behaviour and heavy breathing from late spring to late summer. The temperature-humidity index (THI) was calculated from measurements of 8 barn sensors. We found that, above a THI of 84, cows in advanced pregnancy (>90 days) spent more time breathing heavily and less time eating and in low activity than other cows, while cows in early pregnancy (≤90 days) spent less time breathing heavily, more time eating and in low activity. Cows with 3+ lactations showed less time breathing heavily and in high activity and more time ruminating and in low activity than cows with fewer lactations. Although lactation stage interacted significantly with THI on time spent breathing heavily, ruminating, eating, and in low activity, there was no clear indication at which lactation stage cows were more sensitive to heat. These findings show that cow factors affect the cow’s physiological and behavioural response to heat, which could be used to provide group-specific heat abatement strategies, thereby improving heat stress management.

**Supplementary Information:**

The online version contains supplementary material available at 10.1007/s00484-023-02496-2.

## Introduction

Livestock production is under constant pressure to adapt to a changing climate. Increasingly extreme weather conditions have a major impact on the welfare and production of livestock (Henry et al. 2018). In the case of dairy cattle, this impact is mainly seen in the form of heat stress (Cheng et al. 2022). Heat stress is defined as “the sum of the external forces that act on an animal and cause an increase in body temperature and a physiological response” (Hoffmann et al. 2020). The most widely used environmental indicator of heat stress is the Temperature-Humidity Index (THI; Armstrong 1994). Although the THI does not directly measure the individual burden of the animal, also referred to as “heat load” (Hoffmann et al. 2020), it is closely associated with animal-based indicators, such as body temperature and respiratory rate (Hoffmann et al. 2020; Shu et al. 2021). Various studies have established THI thresholds ranging between 65 and 76 at which the respiratory rate of dairy cows starts to rapidly increase (Shu et al. 2021).

Heat stress affects the milk production and fertility of cows and increases the risk of retained placenta, lameness, mastitis, and even mortality (Kadzere et al. 2002; Galán et al. 2018). It also affects cow behaviour and welfare (Herbut et al. 2021). For instance, cows lie down less during hot weather, and instead prefer to rest while standing to expose more surface area for heat loss (Cook et al. 2007; Allen et al. 2015). Heat stress is also known to reduce dry matter intake (West 2003; Collier et al. 2017) and rumination time (Moretti et al. 2017; Müschner-Siemens et al. 2020). Since rest and nutrient balance are important determinants for cow welfare, health, fertility, and milk production (Esposito et al. 2014; Tucker et al. 2021), these alterations in behaviour have far-reaching implications. Measuring the behaviour of cows is therefore an important instrument to detect early signs of reduced welfare. Due to the increasing industrialization of livestock production, increasing automatization in the monitoring of animal production, welfare, and health, also referred to as Precision Livestock Farming, is warranted (Halachmi et al. 2019). Accordingly, many technologies, usually based on wearable accelerometer-based sensors, have been developed to monitor cow behaviour (Lee and Seo 2021; Riaboff et al. 2022). Recently, a commercial accelerometer-based system (HR-LDn; SCR Engineers Ltd., Netanya, Israel) integrated an algorithm to detect heavy breathing alongside motions related to eating, ruminating, and activity. Heavy breathing is characterized by forward-backward heaving, accelerated respiratory rate, and increased thoracic wall extension and the system detects this based on the magnitude, rhythm, and direction of the involved motions (Bar et al., 2019). Studies that tested the system’s performance found high correlations with vaginal temperature and observations of panting but noted that the system had low sensitivity and was less accurate at detecting lower panting levels (PS = 1; Bar et al. 2019; Islam et al. 2020; Islam et al. 2021).

Cooling strategies are generally applied on the barn level to reduce heat load (Ji et al. 2020). However, cows are often grouped according to lactation stage and/or parity (Contreras-Govea et al. 2015), offering possibilities for specific cooling measures per groups of cows. Indeed, cow factors, such as breed, coat colour, milk yield, and parity, affect the susceptibility of cows to heat stress (West 2003; Galán et al. 2018). For instance, since milk production is accompanied with metabolic heat production, lactating cows, especially high yielding cows, suffer more during heat (West 2003). Related to this, lactation stage was also found to affect heat stress susceptibility (Abeni and Galli 2017; Yan et al. 2021). Mid lactation may be the most sensitive period since milk production in early lactation relies less on food intake and therefore involves less heat production (Abeni et al. 2007; Abeni and Galli 2017). Increasing parity also causes increases in milk yield loss and respiration rate during heat stress, which is probably due to the higher milk yield of multiparous cows (Bernabucci et al. 2014; Yan et al. 2021). Although less studied, cow factors were also found to affect the behavioural response to heat. For instance, multiparous cattle were found to decrease their lying time as THI increased, while primiparous cows did not (Stone et al. 2017). Müschner -Siemens et al. (2020) also found that during heat stress the rumination time decreased strongest in case of late lactation, multiparous, and late gestation cows.

The aim of this work was to study the effect of cow factors (parity, lactation stage, and reproductive status) on the heavy breathing and behavioural response (as measured by a commercial accelerometer system) to hot weather conditions. For this we analysed the behavioural data that was collected by a commercial accelerometer-based system over 4 months from late spring to late summer. We hypothesized that cows with higher metabolic heat (i.e. multiple parities, mid-lactation, and pregnant) would show more time breathing heavily (respiratory rate ≥80 bpm; as measured by a commercial system) and more behavioural responses, e.g. reduced feeding time during hot weather conditions than cows with lower metabolic heat.

## Methods

### Animals and housing

The study was performed on a commercial dairy cattle farm located in the province of Cremona in Northern Italy. This farm has about 115 lactating Italian-Holstein cows, kept in a loose-housing system with free stalls. The subjects were 48 lactating cows (mean parity = 2.2 lactations, range = 1-6 lactations; mean days in milk at start of study = 107, range =20-226; mean milk yield = 39.77 ± 0.32 kg). The subjects were chosen based on days in milk (DIM), preferring cows with less DIM, and presence in the feeding stalls during the mounting of the collars. All subjects were housed in the same monitored building which had three lines of cubicles. The building is open to all sides and has an insulated roof with a ridge opening, high volume low speed fans above the lying area and sprinklers above the feeding area. The cooling systems operated above 26°C (8:00 - 22:00 h; ventilators: continuously; sprinklers: 1 minute every 5 minutes). The building is divided into two sections, one section of 305 m^2^ with on average 35 (primarily primiparous/early lactation) cows and one section of 607 m^2^ with on average 80 cows (all other cows). Feed is supplied at 8:30, and milking at 8 pm and 8 am. From June till August, at approx. 1-week intervals, the individual milk yield during the morning milking session was manually recorded. This value was converted to kg and a daily milk yield value was calculated using the formula: daily milk yield = 2.0 x measured milk yield + 0 x (DIM-158) (based on a 12-hr interval; ICAR 2017). Records of reproductive and health events were used to determine the DIM and the reproductive status for each day of the study. Days on which a subject received reproductive interventions (e.g. insemination or fertility treatment) were excluded from the data. In case a cow was diagnosed to be ill or injured, the period from one week before to one week after was excluded from the data. This led to the exclusion of 449 data points from 39 cows.

### Climate data acquisition

The microenvironmental conditions in the barn where all subjects were housed were measured with eight custom-made sensor nodes, which measure air temperature (°C), relative humidity (%) and light intensity (lx; IBT Systems s.r.l., Milan, Italy). The sensors were placed in different sections of the barn at a height of approximately 1 m above the cow’s head. Data was transmitted every 10 minutes via a 2.4 GHz radio channel to a gateway, which then transmitted the data to the cloud.

### Sensor mounting and behavioural data acquisition

At the start of the experiment, 60 cows were fitted with collars with accelerometer-based sensors (Allflex C-sense™ Flex Tag, SCR Engineers Ltd., Netanya, Israel). The algorithm of the sensors categorizes the accelerometer measurements every minute into 6 mutually exclusive behaviours: ruminating, eating, heavy breathing, low activity/rest, mid activity, and high activity (supplementary materials, table S1). If the behaviour could not be assigned to any category it was recorded as “undefined”. The collars were kept on the cows from mid-April to the end of August (138 days). Twelve cows were sold or dried off before the end of the experiment, resulting in a sample size of 48 cows. After mounting, the system needed 5 days to gather enough information to allow for a correct classification of the different behaviour categories. Therefore, data was only included from 05/01/2021.

### Data processing

Hourly means were calculated for the air temperature, relative humidity, and light intensity. Outliers in the climate data were detected using the Interquartile Range (IQR) method. This was applied for each hour, based on the eight sensor measurements. In addition, measurements that differed by more than 10% from the next lowest/highest measurement were also excluded. If for one hour four or more sensors had to be excluded, the entire hour was excluded from analyses. Combined with occasional power outages and other technical problems, this resulted in 2190 hourly data points. The temperature-humidity index (THI) was calculated from hourly air temperature (AT) and relative humidity (RH) using the formula: THI = (1.8 x AT + 32) - [(0.55 - 0.0055 x RH) x (1.8 x AT -26)] (NRC, 1971). For daily measures the maximum hourly THI of the day was used (Kappes et al., 2022) and divided into six categories (Moretti et al. 2017): 1 (safe): THI < 68, 2 (mild discomfort): 68 ≤ THI < 72, 3 (discomfort): 72 ≤ THI < 75, 4 (alert): 75 ≤ THI < 79, 5 (danger): 79 ≤ THI < 84, and 6 (emergency): THI ≥ 84. For the calculation of daylight hours, the number of hours with a light intensity above 23 lx, which was the maximum level recorded from the artificial lights during the night, were calculated for each day. The manufacturer of the accelerometer-based sensors (SCR Engineers Ltd., Netanya, Israel) provided hourly data, which indicated the number of minutes per hour each cow was involved in one of the six defined behavioural categories (Table S1). Daily means were calculated if at least 22 hours had data (this meant excluding 9 data points, i.e. daily values x cow). For each experiment day, the cows were classified into three levels of parity (1, 2, and 3+), lactation stage (early: <150 DIM, mid: 150-220 DIM, late: >220 DIM), and reproductive status (not pregnant, early pregnancy: ≤90 days, and advanced pregnancy: >90 days). In the end, 5219 data points (daily values x cow) were analysed.

### Statistical analyses

Statistical analyses were performed in SAS version 9.4 (SAS Institute Inc., Cary, NC, USA). General linear mixed models were used to analyse the effects of the THI category on the measured parameters (GLIMMIX procedure; distribution: normal; link function: identity). In this analysis, THI category, parity, parity x THI category, reproductive status, reproductive status x THI category, lactation stage, lactation stage x THI category, and daylight hours were included as fixed effects. The day was included as a random effect and cow was included as the subject. Pairwise comparisons between THI categories, and cows of different parity, reproductive status, and lactation stage were made with the Tukey–Kramer test, using the SLICE option. To determine the threshold at which the effect of THI on heavy breathing increases significantly compared to below the threshold, a piecewise regression analysis was performed following the method of Ryan and Porth (2007). For this the breakpoint was estimated, by viewing the smoothed data using the LOESS procedure. Next, two separate linear regression analyses (REG procedure) were run for the data below and above the estimated breakpoint to estimate the initial intercept and linear functions below and above the breakpoint. Finally, the NLIN procedure (maxiter=1000; method=Marquardt) was run with these estimates. For comparison a simple linear regression (REG procedure) and power regression (NLIN procedure; maxiter=1000; method=Marquardt; model: y = a1 + b1*X**b2) were run on the same data set. This procedure was performed for the entire data set as well as for the different cow groups.

## Results

### General

During the experimental period all the 6 categories of THI were recorded (supplementary materials, Fig. S1). THI category had a significant effect on all measured parameters (all p <0.001; supplementary materials, Table S2). The Tukey-Kramer multiple comparison results are shown in the supplementary materials (Table S3). For time spent breathing heavily, piecewise linear regression estimated a breakpoint (c) at THI = 84.37, with intercept (a) = -3.92, the slope below the breakpoint (b1) = 0.06 and the slope above the breakpoint (b2) = 0.74 (Fig. S2). The mean square of errors for the piecewise regression were 3.68, which was lower than for simple linear regression (4.20), while power regression failed to converge, indicating that the piecewise regression is the best fit for this data. The mean milk yield for each group of cows is shown in the supplementary materials (Table S4).

### Reproductive status

**Fig. 1:**
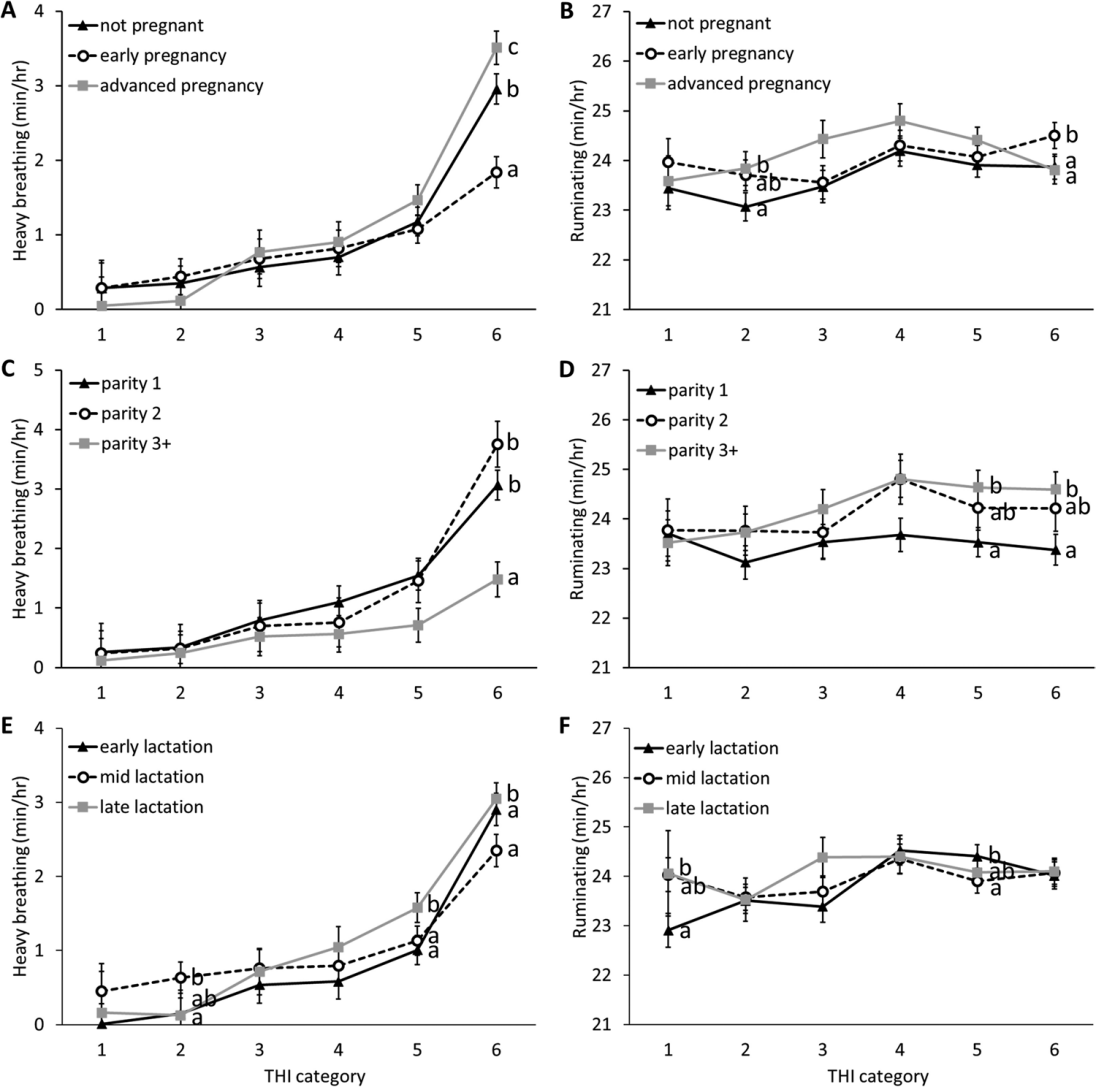
Graphs of effects of daily maximum temperature-humidity (THI) categories on the time spent breathing heavily (left) and ruminating (right), depending on cow factors. The time spent breathing heavily and ruminating is shown for reproductive status (**A** and **B** respectively), parity (**C** and **D** respectively) and lactation stage (**E** and **F** respectively). ^a,b,c^ Groups that differed significantly from each other at the same THI category are identified with different letters. The THI categories are: 1: safe (<68), 2: mild discomfort (68-72), 3: discomfort (72-75), 4: alert (75-79), 5: danger (79-84) and 6: emergency (>84)

**Fig. 2:**
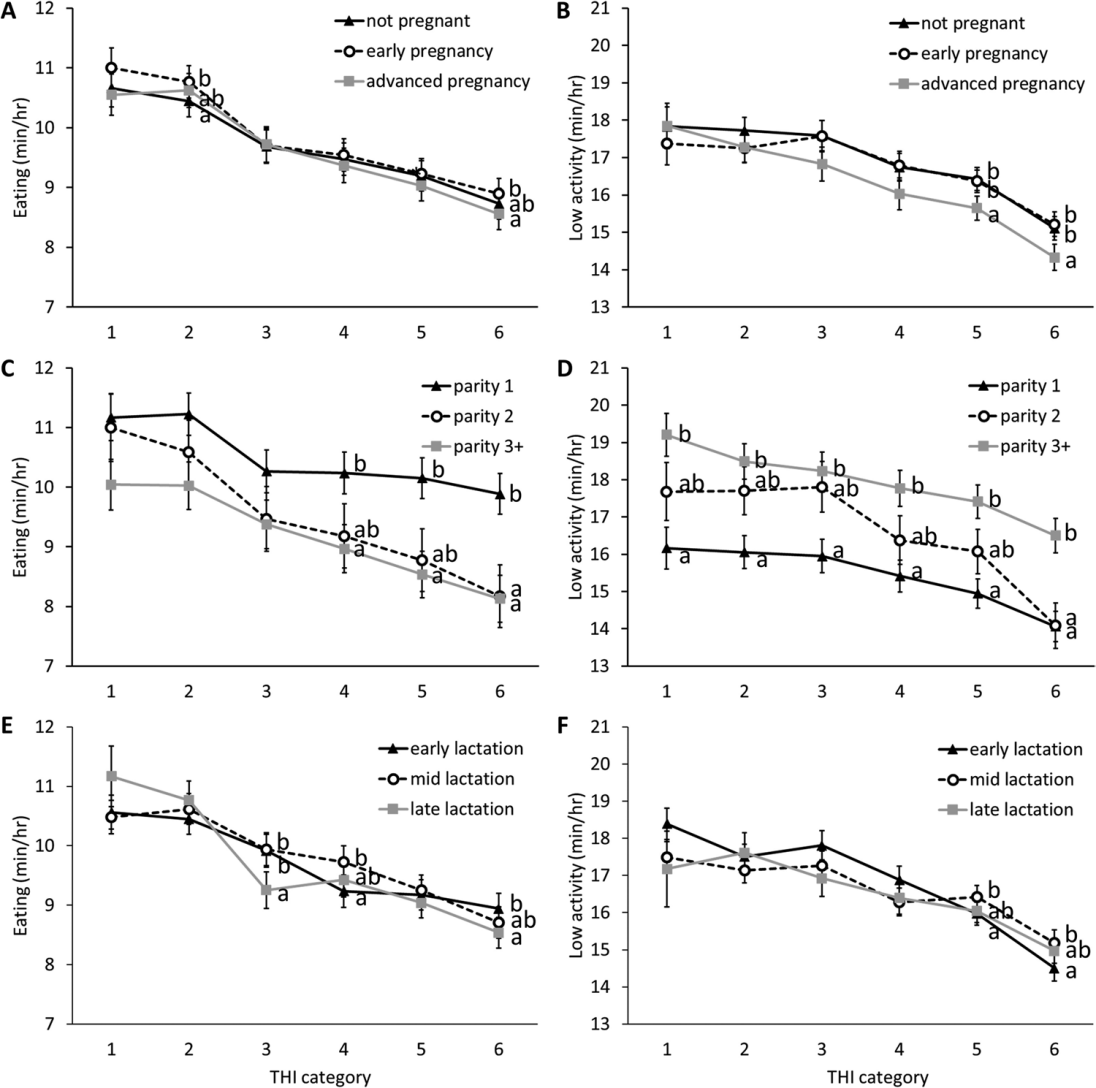
Graphs of effects of daily maximum temperature-humidity (THI) categories on the time spent eating and in low activity, depending on cow factors (reproductive status, parity and lactation stage). The time spent eating and in low activity are shown for reproductive status (**A** and **B** respectively), parity (**C** and **D** respectively) and lactation stage (**E** and **F** respectively). ^a,b^ Groups that differed significantly from each other at the same THI category are identified with different letters (Tukey-Kramer test). The THI categories are: 1: safe (<68), 2: mild discomfort (68-72), 3: discomfort (72-75), 4: alert (75-79), 5: danger (79-84) and 6: emergency (>84)

**Fig. 3:**
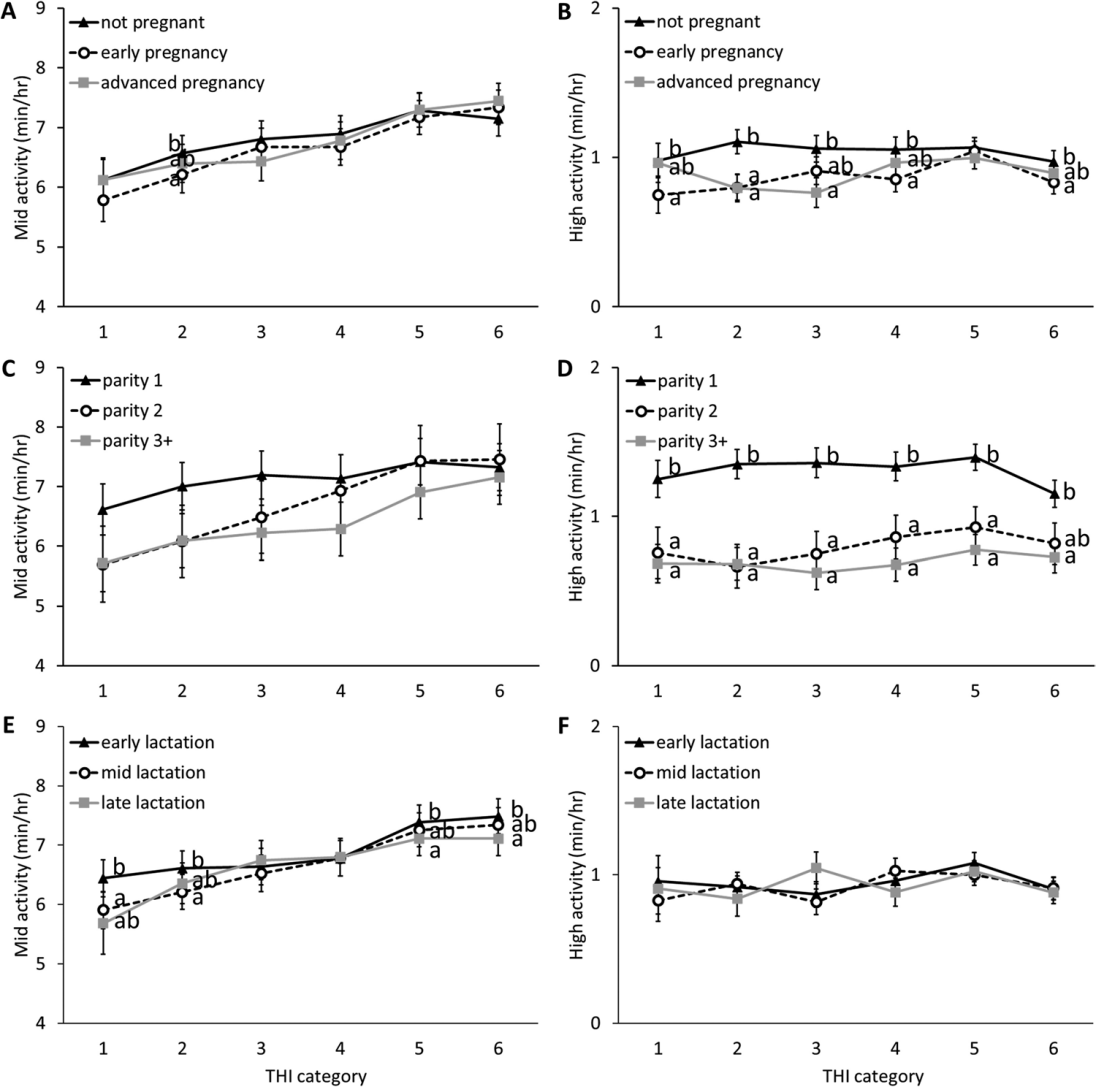
Graphs of effects of daily maximum temperature-humidity (THI) categories on the time spent in mid- and high activity, depending on cow factors (reproductive status, parity and lactation stage). The time spent in mid- and higher activity are shown for reproductive status (**A** and **B** respectively), parity (**C** and **D** respectively) and lactation stage (**E** and **F** respectively). ^a,b^ Groups that differed significantly from each other at the same THI category are identified with different letters. The THI categories are: 1: safe (<68), 2: mild discomfort (68-72), 3: discomfort (72-75), 4: alert (75-79), 5: danger (79-84) and 6: emergency (>84)

Reproductive status had a significant effect on the time spent eating (*p* = 0.019) and in high activity (*p* < 0.001), and a significant interaction with THI on the time spent breathing heavily (*p* < 0.001), ruminating (*p* = 0.004), in mid activity (*p* = 0.019), and in high activity (*p* < 0.001; supplementary materials, Table S2). Tukey-Kramer pairwise comparisons revealed that at THI category 5, cows in advanced pregnancy spent more time breathing heavily than cows in early pregnancy (*p* = 0.015; Fig. 1) and at category 6, cows in advanced pregnancy spent more time breathing heavily than non-pregnant cows (*p* < 0.001) and cows in early pregnancy (*p* < 0.001), while non-pregnant cows still spent more time breathing heavily than cows in early pregnancy (*p* = 0.018). At category 2, cows in early pregnancy spent more time ruminating than non-pregnant cows (*p* = 0.019) and at category 6, cows in early pregnancy spent more time ruminating than both non-pregnant cows (*p* = 0.015) and cows in advanced pregnancy (*p* = 0.011). At category 2, early- pregnant cows spent more time eating than non-pregnant cows (*p* = 0.031) and at category 6, cows in early pregnancy spent more time eating than cows in advanced pregnancy (*p* = 0.027; Fig. 2). At category 5 and 6, cows in advanced pregnancy spent less time in low activity than both early pregnant cows (category 5: *p* = 0.003; category 6: *p* = 0.005) and non-pregnant cows (category 5: *p* = 0.012; category 6: *p* = 0.036). At category 2, cows in early pregnancy spent less time in mid activity than non-pregnant cows (*p* = 0.012; Fig. 3). Non-pregnant cows spent more time in high activity than cows in early pregnancy at categories 1, 2, 4, and 6 (all *p* < 0.036) and more than cows in advanced pregnancy at categories 2 (*p* = 0.002) and 3 (*p* = 0.029). Cows in early pregnancy had the highest breakpoint (THI = 85.96), with the lowest value of time spent breathing heavily (0.84 min/hour) at the breakpoint (Table 1). Non-pregnant cows had the lowest breakpoint (THI = 83.59; Table 1).

**Table 1:** Stepwise regression results for all cows as well as per cow group (grouped according to reproductive status, parity or lactation stage). A indicates the intercept, b1 indicates the slope below the break point, c indicates the breakpoint (temperature-humidity index) and b2 indicates the slope above the breakpoint. Heavy breathing indicates the measured heavy breathing value at the breakpoint (c). DIM = days in milk

Group	A	b1	c	b2	Heavy breathing (min./hour)
All	-3.92	0.06	84.37	0.74	1.25
Not pregnant	-3.35	0.05	83.59	0.68	1.07
Early pregnancy (≤ 90 days)	-1.77	0.03	85.96	0.64	0.84
Advanced pregnancy (> 90 days)	-7.09	0.11	84.72	0.99	2.11
Parity 1	-5.09	0.08	84.35	0.81	1.55
Parity 2	-4.97	0.08	83.78	0.95	1.43
Parity 3+	-1.78	0.03	84.86	0.56	0.76
Early lactation (< 150 DIM)	-3.94	0.06	84.21	1.45	1.24
Mid lactation (150 – 220 DIM)	-2.41	0.04	84.45	0.53	0.90
Late lactation (≥ 220 DIM)	-4.99	0.08	85.96	1.03	1.77

### Parity

Parity had a significant effect on the time spent eating (*p* = 0.042), in low activity (*p* < 0.001), and in high activity (*p* < 0.001), and a significant interaction with THI on all parameters (all *p* <0.0066; supplementary materials, Table S2). Tukey-Kramer pairwise comparisons revealed that at THI category 6, cows with 3+ lactations spent less time breathing heavily than cows in their 1^st^ or 2^nd^ lactation (vs. parity 1: *p* < 0.001; vs. parity 2: *p* < 0.001; Fig. 1). Cows with 3+ lactations also spent more time ruminating than primiparous cows at THI categories 5 (*p* = 0.039) and 6 (*p* = 0.026). Conversely, primiparous cows spent more time eating than cows with 3+ lactations at categories 4, 5, and 6 (all *p* < 0.046) and more than cows in their 2^nd^ lactation at category 6 (*p* = 0.018; Fig. 2). Cows with 3+ lactations spent more time in low activity than primiparous cows at all THI categories (all *p* < 0.002) and more than cows in their 2^nd^ lactation at category 6 (*p* = 0.005). Primiparous cows spent more time in high activity than cows with 3+ lactations on all THI categories (all *p* < 0.006; Fig. 3) and more than cows 2 lactations at categories 1 to 5 (all *p* < 0.020). Although piecewise regression results did not differ much between parities, cows with 3+ lactations had the highest breakpoint (THI = 84.86) and cows with 2 lactations had the lowest breakpoint (THI = 83.78; Table 1).

### Lactation stage

Lactation stage had a significant effect on the time spent in mid activity (*p* = 0.019), and a significant interaction with THI on the time spent breathing heavily (*p* = 0.001), ruminating (*p* = 0.006), eating (*p* < 0.001), and in low activity (*p* = 0.006; supplementary materials, Table S2). Tukey-Kramer pairwise comparisons revealed that at THI category 2, mid-lactation cows spent more time breathing heavily than early-lactation cows (*p* = 0.047), while at category 5, late-lactation cows spent more time breathing heavily than early- and mid-lactation cows (vs. early: *p* = 0.002; vs. mid: *p* = 0.002; Fig. 1) and at category 6, mid-lactation cows spent less time breathing heavily than early- and late-lactation cows (vs. early: *p* = 0.002; vs. late: *p* < 0.001). Mid-lactation cows ruminated more than early-lactation cows at category 1 (*p* = 0.019), while the inverse effect was found at category 5 (*p* = 0.003). At category 3 late-lactation cows ate less than early- and mid-lactation cows (vs. early: *p* = 0.023; vs. mid: *p* = 0.004), while at category 4, mid-lactation cows ate more than early-lactation cows (*p* = 0.005) and at category 6, early-lactation cows ate more than late-lactation cows (*p* = 0.016; Fig. 2). Mid-lactation cows rested more than early-lactation cows at categories 5 (*p* = 0.035) and 6 (*p* = 0.019). Early-lactation cows spent more time in mid activity than mid-lactation cows at categories 1 (*p* = 0.041) and 2 (*p* = 0.015) and more than late-lactation cows at categories 5 (*p* = 0.049) and 6 (*p* = 0.028; Fig. 3). Late-lactation cows had the highest breakpoint (THI = 85.96), and early-lactation cows had the lowest breakpoint (THI = 84.21; Table 1).

## Discussion

Our findings of negative effects of THI on time spent eating and in low activity (resting) and positive effects on time spent in mid activity are in line with previous findings based on the same system (Ramón-Moragues et al. 2021; Kappes et al. 2022). In contrast to these previous reports though, we found that rumination time slightly increases until THI category 4. This is difficult to explain, since rumination time is dependent on dry matter intake (Moallem et al. 2010) and, therefore, should also have decreased. The breakpoint for heavy breathing time was estimated at a THI of 84, indicating that this measure starts to increase at the emergency level (Moretti et al. 2017). Since, cooling systems were operational, this may have reduced the heat load for the subjects and thereby increased the breakpoint. Our findings are, however, in line with previous reports (Islam et al. 2020; Islam et al. 2021) which showed that the heavy breathing measure is more an alarm for serious health risks due to heat exposure, rather than an early indicator.

As expected, we found that at higher THI categories cows in advanced pregnancy spent more time breathing heavily and less time eating and in low activity, suggesting they were most sensitive to heat. This could be due to increasing heat production as gestation progresses (Ferrell et al. 1976). In contrast to a previous report (Müschner-Siemens et al. 2020) the reduction in rumination time was not so clear in advanced pregnancy, but since the general rumination pattern during hot weather conditions also differed, it is difficult to compare the two findings. Surprisingly, we found that cows in early pregnancy seemed less affected by high THI values than both non-pregnant and cows in advanced pregnancy, as evidenced by a lower rise and higher THI breakpoint in heavy breathing time and more time spent ruminating, eating, and resting at higher THI categories. Although metabolic heat production due to gestation is still low during early pregnancy (Ferrell et al. 1976), the increase in progesterone, which happens at the start of pregnancy (Butler et al. 1996), is found to increase body temperature (Suthar et al. 2012). This means that early pregnant cows were expected to have more heat to dissipate (and therefore to suffer more during hot weather conditions) than non-pregnant cows. Regarding rumination time, our findings confirm previous findings that recently inseminated cows ruminate longer than non-pregnant cows (Antanaitis et al. 2020). However, more research is needed to understand the effects of gestation on behaviour and heat stress susceptibility.

Also contrary to our expectations, at higher THI categories, cows with 3+ lactations spent less time breathing heavily and in high activity and more time ruminating and in low activity than cows with fewer lactations, suggesting that these cows are less affected by hot weather conditions. This contradicts previous reports that multiparous cows are more susceptible to heat stress (Bernabucci et al. 2014; Yan et al. 2021), while other studies found no effect of parity on rumination during hot weather (Soriani et al. 2013; Moretti et al. 2017). Indeed, considering that the primiparous cows had slightly lower milk yields (supplementary materials, Table S4), they would have had less metabolic heat produced from milk synthesis (West 2003). Although the method to calculate daily milk yield is established (ICAR 2017), small inaccuracies may exist since afternoon milk yields are usually lower because of diurnal variation (Quist et al. 2008). However, since diurnal effects can be assumed to be similar across the tested groups, this should not affect the general interpretations. We also found that, unlike multiparous cows, primiparous cows did not reduce eating time at higher THI categories as a strategy to reduce heat production (Renaudeau et al. 2012). This group also spent more time in high activity and less time in low activity than cows with 3+ lactations. This is in line with previous findings (Stone et al. 2017) and may be related to higher stress levels (González et al. 2003) caused by the environmental and social changes that primiparous cows experience after their first calving (Soriani et al. 2012).

Although we found significant interactions between lactation stage and THI categories on most of the measured parameters, the results provide no clear pattern. Late-lactation cows spent more time breathing heavily at higher THI categories, suggesting they are most sensitive to heat at this stage, which is odd, because they had the lowest milk yield (supplementary materials, Table S4). However, this interpretation is only further supported by the less time late-lactation cows spent eating at THI category 6. In contrast, early lactation cows spent less time in low activity and more time in mid activity at higher THI categories and had the lowest breakpoint. Therefore, it is difficult to infer a clear effect of lactation stage on the sensitivity to heat, which contrasts with previous findings that indicated that mid-lactation cows are most sensitive to heat (Abeni et al. 2007; Abeni and Galli 2017). It is therefore important that the effects of lactation stage on heat stress susceptibility are further studied.

Overall, these findings show that cow factors affect both the automatically measured heavy breathing and behavioural response to heat in dairy cattle, which may be used to improve heat abatement strategies. The differences in breakpoints did not vary much between the different groups. However, considering that these breakpoints signify emergency THI categories, these differences could still prove vital. Grouping cows based on parity or lactation stage is already a common practise since it reduces competition and increases milk yield (Contreras-Govea et al. 2015). Our findings show that such group divisions (as well as a division based on reproductive status) also could benefit heat abatement as this would allow to start cooling measures, such as the activation of the sprinklers or ventilation, at different THI thresholds. In addition, more heat sensitive groups, i.e. advanced pregnant cows and cows with less than 3 lactations, could be kept in sections of the farm that are better structured to benefit heat abatement, such as with larger lateral openings to improve air circulation and a E-W/NE-SW orientation (Lovarelli et al. 2021). Our findings also show that cow factors affect the behavioural response to hot weather conditions, which could also be used to improve heat stress management. For instance, multiparous cows substantially reduced their eating time, which negatively affects milk yield and ultimately leads to negative energy balance and lowered body condition scores (Polsky and von Keyserlingk 2017). This could be prevented by changing feeding regimes or diet composition to improve feed efficiency (Sammad et al. 2020). On the other hand, cows in advanced pregnancy and in their 1^st^ or 2^nd^ lactation are more likely to reduce their time spent in low activity, which increases the risk of lameness and other diseases (Cook et al. 2007; Tucker et al. 2021). For such groups it may be more beneficial to improve climatic conditions in the cubicle, for instance by using bedding that benefits heat dissipation from the cow, e.g. wood shavings (De Palo et al. 2006), or by installing ventilators above the cubicles (Calegari et al. 2014).

To conclude, our findings show that parity and reproductive status clearly affect both the automatically measured heavy breathing and behavioural response of dairy cattle to heat, while the effect of lactation stage was less straightforward. This shows that keeping cows in different groups according to their reproductive status, parity or lactation stage could benefit heat stress management through the adoption of group-specific heat abatement strategies.

## Supplementary Information

ESM 1 (PDF 314 kb)
