# Supplementary material for: Effects of cow reproductive status, parity and lactation stage on behaviour and heavy breathing indications of a commercial accelerometer during hot weather conditions
Source: Int J Biometeorol. 2023 May 29;67(7):1263–72. doi: 10.1007/s00484-023-02496-2 (PMC10329063; doi:10.1007/s00484-023-02496-2)
Supplement: Supplementary file 1 — (PDF 314 kb) [file 484_2023_2496_MOESM1_ESM.pdf]

Supplementary materials

Table S1: Description of the behaviours classified by the commercial collars (Allflex® DataFlow II, Monitoring Neck Tag, SCR Engineers Ltd., Netanya, Israel)

Animal State	Description
Low Activity - Resting	Standing, lying still, or sleeping, and *not* ruminating. Head/neck may be in any orientation relative to body, providing they are stationary except for occasional, very brief movements as might occur when changing position during sleep
Mid Activity	Non-specific behaviour where the animal exerts energy. For example, wandering about with irregular pace or pattern or standing and performing behaviours such as sniffing, scratching, searching for feed, and social interactions. This is a "last resort" bin used only when the animal is not performing any of the other discrete behaviours
High Activity	Non-specific behaviour where the animal exerts energy. This includes any combination of energy-intensive behaviours such as moving at a pace faster than a walk, leaping, and buck-kicking, even if the activity is relatively short and does not occupy an entire minute
Rumination	Rhythmic circular/side to side movements of jaw not associated with eating, interrupted by brief (< 5 sec) pauses during time that bolus is swallowed, followed by continuation of rhythmic jaw movements. If this is detected, the cow is recorded as ruminating, regardless of activity status.
Eating	Muzzle/tongue physically contacts and manipulates feed, often but not always followed by chewing. The cow may move from one location to another while eating.
Heavy Breathing	This is recorded when the respiratory rate is above 80 bpm, with easily visible lateral and forwards-backwards chest expansion/body/abdominal movements when breathing. May include occasional or regular open mouth (space between lips visible). This is not recorded if the cow is also ruminating. The neck and head may be extended.

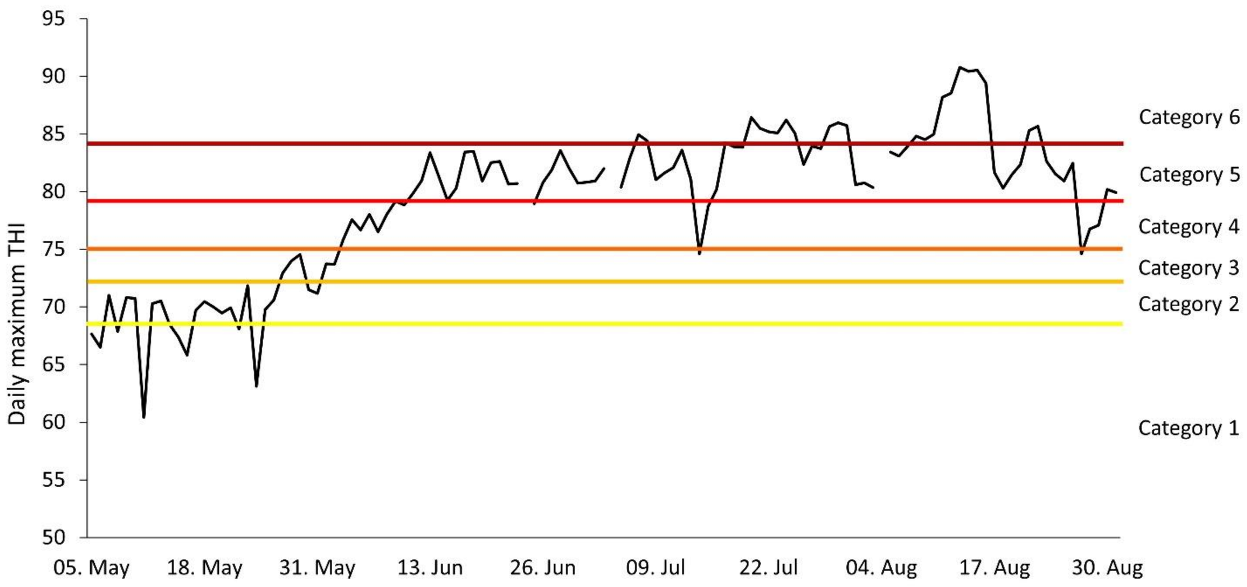

Fig. S1: Maximum daily temperature-humidity index (THI) values during the experimental period. The coloured lines indicate the thresholds of the different THI categories: 1: safe (<68), 2: mild discomfort (68-72), 3: discomfort (72-75), 4: alert (75-79), 5: danger (79-84) and 6: emergency (>84).

Table S2: Results of the GLIMMIX models. F and p values are given for each fixed effect included in the model and for all measured accelerometer parameters. The category of the temperature-humidity index (THI) is based on maximum daily values: 1: safe (<68), 2: mild discomfort (68-72), 3: discomfort (72-75), 4: alert (75-79), 5: danger (79-84) and 6: emergency (>84).

	Heavy breathing		Ruminating		Eating		Low activity		Mid activity		High activity	
Effect	F	p	F	p	F	p	F	p	F	p	F	p
THI category	136.70	<0.001	5.41	<0.001	108.02	<0.001	57.21	<0.001	43.74	<0.001	8.19	<0.001
Parity	1.45	0.246	1.57	0.220	3.41	0.042	9.04	<0.001	0.76	0.477	11.82	<0.001
Reproductive status	2.65	0.071	2.99	0.050	3.99	0.019	2.40	0.091	2.38	0.092	9.89	<0.001
Lactation stage	1.13	0.324	0.76	0.465	0.79	0.455	0.89	0.413	3.97	0.019	0.30	0.744
THI category*parity	13.69	<0.001	2.45	0.007	10.33	<0.001	3.76	<0.001	10.11	<0.001	3.89	<0.001
THI category*reproductive status	8.25	<0.001	2.61	0.004	0.99	0.449	0.83	0.603	2.13	0.019	3.13	<0.001
THI category*lactation stage	2.94	0.001	2.45	0.006	3.05	<0.001	2.48	0.006	1.30	0.227	1.50	0.133
Hours of daylight	55.86	<0.001	3.48	0.062	4.00	0.046	4.51	0.034	96.51	<0.001	178.28	<0.001

Table S3: Least square means ± standard error of measured parameters at the different temperature -humidity index (THI) categories: 1: safe (<68), 2: mild discomfort (68-72), 3: discomfort (72-75), 4: alert (75-79), 5: danger (79-84) and 6: emergency (>84). ^{a,b} means with different superscripts in the same row differ significantly (Tukey-Kramer test).

	THI category					
Parameter	1	2	3	4	5	6
Heavy breathing	0.21 ± 0.29 ^{ab}	0.30 ± 0.21 ^{a}	0.67 ± 0.20 ^{ab}	0.81 ± 0.19 ^{b}	1.24 ± 0.18 ^{c}	2.77 ± 0.18 ^{d}
Ruminating	23.67 ± 0.37 ^{abc}	23.54 ± 0.26 ^{a}	23.82 ± ^{ab}	24.43 ± 0.24 ^{c}	24.13 ± 0.21 ^{bc}	24.06 ± 0.22 ^{bc}
Eating	10.74 ± 0.29 ^{d}	10.61 ± 0.26 ^{d}	9.70 ± 0.26 ^{c}	9.46 ± 0.25 ^{c}	9.15 ± 0.25 ^{b}	8.73 ± 0.25 ^{a}
Low activity	17.69 ± 0.45 ^{c}	17.42 ± 0.32 ^{c}	17.33 ± 0.32 ^{c}	16.52 ± 0.31 ^{b}	16.15 ± 0.28 ^{b}	14.88 ± 0.29 ^{a}
Mid activity	6.01 ± 0.32 ^{a}	6.39 ± 0.29 ^{a}	6.64 ± 0.29 ^{ab}	6.78 ± 0.29 ^{b}	7.25 ± 0.28 ^{c}	7.31 ± 0.28 ^{c}
High activity	0.90 ± 0.10 ^{ab}	0.90 ± 0.07 ^{a}	0.91 ± 0.07 ^{a}	0.96 ± 0.07 ^{ab}	1.03 ± 0.06 ^{b}	0.90 ± 0.07 ^{a}

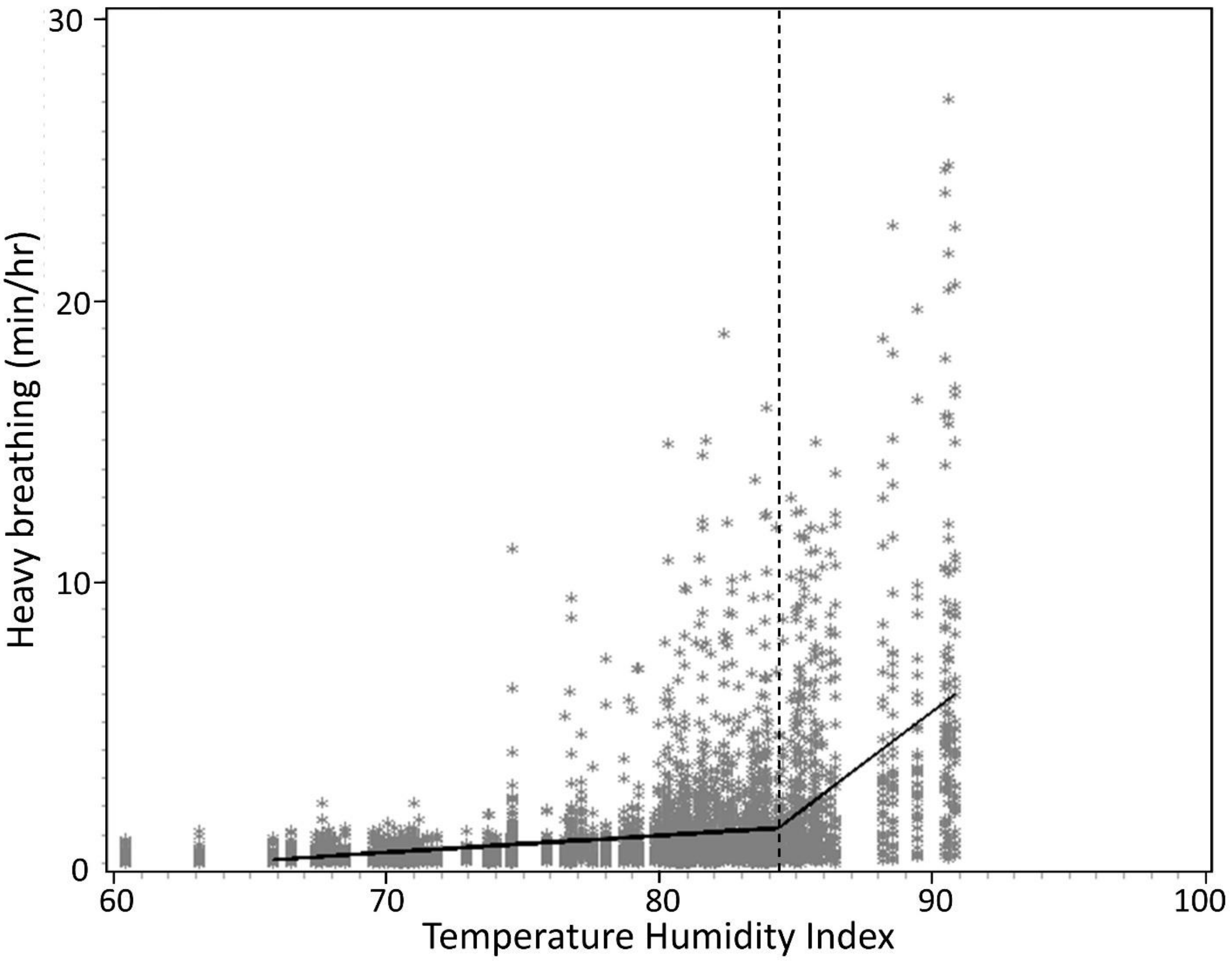

Fig. S2: Results of the stepwise regression of heavy breathing relative to the daliy maximum temperature-humidity index (THI) for the entire group. The breakpoint at which the effect of THI on heavy breathing changes significantly is indicated by the dashed line.

Table S4: LS means and standard error (S.E.) of daily milk yield for each group of cows (grouped according to reproductive status, parity, or lactation stage)

factor	Cow group	Daily milk yield (kg)	S.E.
Reproductive status	Not pregnant	41.19	1.08
	Early pregnancy (≤ 90 days)	40.98	1.03
	Advanced pregnancy (> 90 days)	40.07	1.11
Parity	Parity 1	37.68	1.33
	Parity 2	42.56	2.03
	Parity 3+	42.01	1.54
Lactation stage	Early lactation (< 150 DIM)	41.95	1.05
	Mid lactation (150 – 220 DIM)	40.62	0.99
	Late lactation (≥ 220 DIM)	39.68	1.04
